# Learning from vision-to-touch is different than learning from touch-to-vision

**DOI:** 10.3389/fnint.2012.00105

**Published:** 2012-11-20

**Authors:** Dagmar A. Wismeijer, Karl R. Gegenfurtner, Knut Drewing

**Affiliations:** Allgemeine Psychologie, Justus-Liebig Universität GießenGießen, Germany

**Keywords:** arbitrary association, cue-interaction, gloss, learning, multi-sensory, softness, touch, vision

## Abstract

We studied whether vision can teach touch to the same extent as touch seems to teach vision. In a 2 × 2 between-participants learning study, we artificially correlated visual gloss cues with haptic compliance cues. In two “natural” tasks, we tested whether visual gloss estimations have an influence on haptic estimations of softness and vice versa. In two “novel” tasks, in which participants were either asked to haptically judge glossiness or to visually judge softness, we investigated how perceptual estimates transfer from one sense to the other. Our results showed that vision does not teach touch as efficient as touch seems to teach vision.

## 1. Introduction

Every day we obtain information about our environment using different sensory modalities. To create a unified percept of our environment, information from different senses needs to be combined. We can learn which information about our environment is most likely to belong together by analyzing statistical correlations, and by interacting with the environment. For example, we can learn that larger objects are heavier than smaller ones—given that they have the same visual appearance—by lifting them. Even more so, if we can get estimates of an environmental property from different sensory modalities, we can calibrate the senses with respect to each other and the world. Two important research questions within the field of multi-sensory perception are how such learning happens, that is how information from one sense is transferred to the other and how information from different senses is combined.

Studies on multi-sensory perception provide evidence for the theory of reliability-based cue integration, meaning that information from different senses are weighted according to their reliability before being combined, see Ernst and Bülthoff (2004) for a review.

Some of these studies focused on space perception, e.g., perceived surface orientation and depth, and showed that touch can teach vision (Ernst et al., 2000; Atkins et al., 2001; Ho et al., 2009; van Beers et al., 2011). Ernst et al. (2000), for instance, have shown that haptic feedback correlated with one of two conflicting visual slant cues entices observers to give more weight to that cue when judging slant. Evidence that vision can teach touch, is much thinner and mainly comes from developmental studies (Gori et al., 2008, 2011). From adult studies on stimulus properties such as size—which is thought to be sensed more accurately by the haptic system than the visual system (Gori et al., 2008)—and weight, there is evidence that haptic estimates are influenced by visual cues (Hillis et al., 2002; Flanagan et al., 2008; Buckingham et al., 2009; Walker et al., 2010), i.e., vision can at least influence touch. However, none of these studies have shown that vision can teach touch, in the sense that learning leads to a change in weights given to single cues, as has been shown previously for touch-to-vision learning. Thus, it is unknown whether such transfer of information can occur to the same extent from vision to touch. In this study, we investigated whether vision can teach touch to the same extent as touch seems to teach vision.

A draw-back of stimulus properties used in previous studies, e.g., surface orientation, depth, and size, is that they are naturally sensed by both the haptic and visual system. In addition, one system might be able to do so more accurately, leading to unevenly distributed weighting of visual and haptic cues, i.e., capture by one or the other sense (Ernst and Bülthoff, 2004). The fact that one sense might dominate the other, would make it difficult to study information transfer in both directions. It would thus be ideal to study multi-sensory perception of environmental properties that are naturally sensed by either of the two sensory systems, but not by both.

In such a case, one cannot disturb the natural relation between e.g., the haptic and visual counterparts of a particular property as is usually done in multi-sensory perception studies. But, one could create an artificial correlation between two unrelated sources of information. That one can learn such arbitrary statistical correlations in a relatively short time, has been studied by Ernst (2007). He showed that mandatory perceptual association between brightness and stiffness of an object occurred after exposure to an artificial statistical correlation between the two.

Using artificial correlations, we studied the influence of a non-natural cue on a perceptual estimate, and we did so in both directions: from-vision-to-touch and from-touch-to-vision. To this end, we chose two material properties that are naturally uncorrelated to one another and can only be directly sensed by one modality. As a “haptic material property” we used compliance, which can only be directly sensed by the haptic system (because only the haptic system has direct access to force information) and any visual corrugate has to be learned (Drewing et al., 2008). As a “visual material property” we chose gloss, which has no haptic corrugate.

Real haptic objects with varying compressibility gave participants a haptic cue to the compliance of the stimulus. As a visual cue to gloss, we varied the amount of light that was reflected by a virtual cylindrical object and the size of the highlights on it. Participants were given one stimulus defined by both cues and another defined by one or the other cue and were instructed to discriminate between the two stimuli regarding gloss or softness. For instance, a participant was given one stimulus which was defined by both a compliance and a gloss cue (standard stimulus) and one which was only defined by a compliance cue (comparison stimulus). Now, we could ask the participant to judge one of two material properties: gloss or compliance. The “natural task” would consist of judging “which one feels softer,” in which case the participant could compare the two haptic compliance cues, even before any statistical correlation between the haptic and visual cues was learned. In contrast, in the “novel task,” judging “which one feels less glossy,” the participant should, initially, hardly be able to make a reliable judgment, unless he/she relied on a pre-existing association between felt softness and seen gloss (and transferred his/her perceived softness into some estimate of felt glossiness).

We used a 2 × 2 between-participants design [2 senses or judgment modalities, (haptic or visual) × 2 judged dimensions (gloss or softness)], with a two alternative forced choice task (2-AFC task). In a 2-AFC task, the participant is forced to make a discriminative decision between two stimuli. Such a task makes it possible to measure the point of subjective equality (PSE)—which assesses the stimulus parameter value at which the two stimuli are perceived to be identical—and the just noticeable difference (JND)—which assesses the discrimination threshold. The judgment modality, or sense used to judge a particular stimulus property (dimension), not only refers to how participants had to judge (“feel” or “look”), but also reflects which cues were available. For the haptic sense, there was always a haptic cue for two stimuli (and a visual cue for just one stimulus), whereas for the visual sense, there was a visual cue for two stimuli (and a haptic cue for just one stimulus). We thus had two novel-task conditions (“which one looks softer” and “which one feels less glossy”) and two natural task conditions (“which one feels softer” and “which one looks less glossy”).

Since we could not be a 100% sure that participants did not have some pre-existing association between the two cues, we tested that in an initial (control) session in which we made sure that there was no overall correlation (Pearson's product-moment correlation coefficient was 0) between the two cues. Then in a second session (on another day), participants were first subjected to a short (56 trials) training in which the overall correlation (Pearson's product-moment correlation coefficient) between the two cues was 0.94, followed by training trials interleaved with test trials (the overall correlation was 0.85 in this part).

To make comparisons between experimental conditions easier, we refer in the rest of the manuscript to the cues we used as main and associated cues. The **main cue** always refers to that cue which is naturally sensed by the **judgment modality**, i.e., the haptic compliance cue for haptic judgments (“which stimulus feels …”) and the visual gloss cue for the visual judgments (“which stimulus looks …”). The **associated cue** then refers to the other cue of the standard stimulus, i.e., the visual gloss cue for the haptic judgments and the haptic compliance cue for the visual judgments. In addition, the **judged dimension** relates to the material property participants had to judge: less glossy or softer.

We measured learning of the arbitrary association by changes in the estimation of the PSE and JND as follows: we introduced small discrepancies between the main and associated cue values in our test stimuli.

We predicted for the two natural tasks (haptic softness judgments and visual gloss judgments) that learning the arbitrary association would lead to estimations derived from reliability-based weighting of cues. This means that, before learning, the PSE should only depend on the main cue, because gloss and softness are not (naturally) related to one another. Learning the arbitrary association should lead to a shift in the PSE in the direction of the associated cue value, thus giving some weight to the associated cue in the judgment. In the novel task, the participant should have learned how perceived softness can be transferred into an estimate of gloss before comparing the two stimuli and thus be able to make a reliable judgment of felt gloss. After learning, cues should be combined similarly as in the natural task, leading to a similar shift in the PSE in the direction of the associated cue.

Learning the arbitrary association should lead to a decrease in the JND in all tasks. We predicted that the largest changes would occur in the novel tasks, since we did not expect participants to perform the task very well in the initial session. In addition, we predicted that after learning, the JND should be same for all tasks, given that we tried to have similar JND values for visual and haptic cues. Otherwise, the biggest difference in JND should arise between judgment modalities, but not judged dimensions.

With the experimental paradigm as sketched, we investigated how participants in the novel task learned to transfer perceptual estimates from one sense to another. And, by interchanging both the single cue stimuli (gloss vs. compliance cue only) and the dimension to be judged, we were able to compare learning between touch-to-vision and vision-to-touch transfers. In addition, the natural tasks allowed us to study whether learning arbitrary correlations influenced the integration of the associated cue into a combined percept similarly for vision-to-touch and touch-to-vision.

## 2. Materials and methods

### 2.1. Participants

All 29 participants (three male, mean age 24 years with a standard deviation of 4 years, one left-handed participant, and by accident, one participant more in the haptic soft condition) had normal or corrected-to-normal vision, a stereoacuity of at least 60 arcsec (Randot Stereo Fly, graded circle test) and no sensory or motor deficits. They were either paid for their participation (8 Eur/h) or given credits as part of their psychology curriculum.

### 2.2. Apparatus

**Figure 1:**
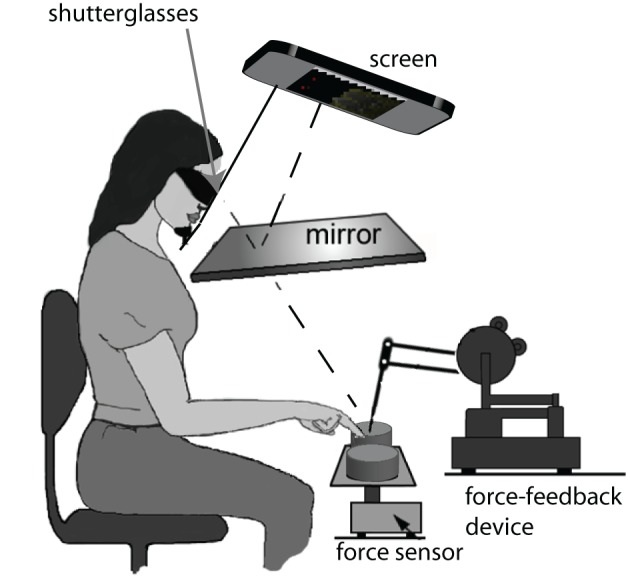
**The experimental setup.** Participants were seated in front of the virtual reality setup, with their heads resting on a chin- and headrest for stabilization purposes. Participants wore shutter glasses and viewed the visual 3D scene via a silver-coated mirror. The real haptic objects were placed in front of the participant on the force sensor. We used a force-feedback device to track the location of the participant's index finger. Participants responses were registered by tracking (with the force-feedback device) which virtual decision button was touched.

**Figure 2:**
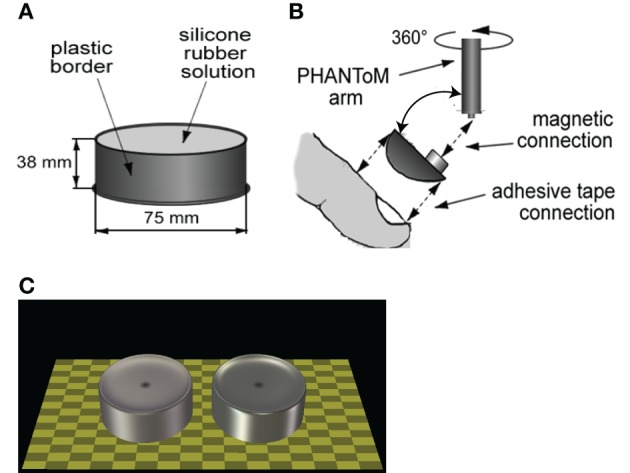
**Stimuli and setup detail. (A)** Sketch of a haptic object. **(B)** Attachment of finger to force-feedback device. **(C)** Sketch of a visual scene showing the two extreme cases of our gloss axis. Note that in reality the visual correlate would only be visible when the participant pressed on the object (or in its presumable location for visual-only comparisons). Also note that the shader has been turned off to generate these images and that the actual scene was dimmer than shown here.

Figure 1 shows a sketch of the experimental setup. Participants sat in front of the setup resting their heads on both a chin- and headrest to minimize head movements. To track the position of the hand, the index finger of participant's preferred hand (participants could be either left or right handed) was attached to a force-feedback device (Phantom Premium 1.5, 1000 Hz, spatial resolution: 0.03 mm), by gluing a reusable plastic finger nail with a small magnet onto the finger (see Figure 2B). The participants viewed the visual scene through a mirror while wearing shutter glasses (NVIDIA, 3D vision kit). The visual scene was displayed on a Samsung Syncmaster 2233RZ (120 Hz) and generated on a DELL Precision 380. Because a mirror rotates polarized light (emitted by any LCD) by 90^°^, we had to rotate the screen by the same amount to realign the polarizing filters of the screen and those of the shutter glasses. Due to the fact that brightness was dependent on the viewing angle along the vertical axis of the LCD screen, we corrected the whole visual scene using shaders (OpenGL/GLSL: Woo et al., 1997; Rost, 2006).

### 2.3. Stimuli

A stimulus consisted of either a haptic compliance cue or a visual gloss cue or a combination of both (standard stimulus). The haptic compliance cue was obtained by pressing onto custom made silicone cylindrical objects, for an example see Figure 2A. They had compliance values of: 0.124, 0.136, 0.148, 0.159, 0.177, 0.189, and 0.198 mm/N, identical to the ones used by Kaim and Drewing (2011). We measured the compliance of each object using a cylindrical probe of 1 cm^2^. The difference between consecutive compliance values was equal to approximately half a JND value (and see Kaim and Drewing, 2011). Instead of referring to the compliance values, we used a JND scale with one unit approximately equal to 0.5 JND (−3 … 3, with 0 equal to a compliance value of 0.159).

The visual gloss cue was conveyed by a virtual 3D cylindrical NURB (non-uniform rational basis spline) surface that was generated with a custom made C(++) program using OpenGL, see Figure 2C. NURB refers to a mathematical technique using polynomials to describe smooth surfaces, which is available in OpenGL (Woo et al., 1997). Using this mathematical technique, we were able to generate virtual cylindrical objects that had a similar non-flat top surface as our real objects, which were slightly convex. The virtual cylinders had the same dimensions (height and diameter) as the real objects. Differences in gloss appearance were established by co-varying two OpenGL defined object parameters: the shininess component, which regulates the size and brightness of specular highlights on a surface, and the specular component, which defines how a material reflects specular light. In order to generate more than one highlight on the virtual cylinders, we used several light sources to illuminate the scene, which were spread symmetrically around the vertical meridian. In a separate pilot experiment (four subjects, magnitude estimation of identical surfaces but with sparser lighting), we determined that perceived glossiness depended linearly on the specular component. In addition, there was an interaction effect between the shininess component and the specular component on perceived gloss, which became non-linear for extreme values of these two components. We stayed within the perceptually linear range and varied the shininess component between 30 and 90 (total range [0, 128]), and the specular component was varied between 0.25 and 0.55 (total range [0, 1]). We then chose a set of parameter values for which the difference between consecutive gloss values was approximately half a JND. We verified this assumption in another pilot experiment (almost identical to the study reported here but with longer visual stimulus exposure times, 10 subjects, 2-AFC task), in which the mean JND was 2.4 with a standard deviation of 2.66. In the current study we found a mean JND of 3.3 with a standard deviation of 2.0. Instead of referring to the gloss parameters, we used a JND scale, with one unit being approximately 0.33 JND (in the current study, range between −3 … 3 and with 0 equal to a specular component of 0.4 and a shininess component of 60).

The virtual cylinders were positioned such that they completely coincided with the haptic objects in real space. These visual correlates were only visible when observers pressed onto the haptic object, otherwise a ring was shown to identify the location of the object. In cases without the haptic object, participants had to place their finger in the empty space where the comparison stimulus was, to make the visual cue visible.

### 2.4. Task and procedure

#### 2.4.1. Task

Before starting the experiment, participants were told that they had to judge which stimulus felt or looked as being softer, or less glossy, respectively. In addition, we told them which type of stimuli would be present in the experiment. For instance, a participant in the haptic soft condition was told that he/she would be able to (and had to) press onto two haptic stimuli, but that only one stimulus would also be shown on the screen. After that, he/she was told to judge which one of the stimuli *felt softer* and then touch the virtual decision button (“fuehlt sich weicher an”) above the softer stimulus. We additionally instructed participants to press in the middle of the haptic objects and not to slide their finger across them [a natural movement made by participants to judge surface roughness (Lederman and Klatzky, 1993)].

They were then given four initial trials, in which we used the stimuli that had the most extreme gloss and/or compliance values. After that the experiment began.

Before each trial, participants were asked to position the finger attached to the force-feedback device in the left-bottom corner of the virtual environment, which was visualized by the word “WARTEN” (wait). This gave the experimenter the opportunity to place the haptic object(s) for the next trial in the designated area(s), without touching the observer. After placement of the stimuli, the next trial started. Participants would then move their index finger (either to the left or the right stimulus) and press on the haptic object—or in the space where it should have been—and thus trigger the visual cue to be shown. They then could either touch the same object again, or move to the other object. After touching each stimulus location, they indicated which stimulus was “less glossy” or “softer” by pressing the corresponding virtual decision button. After that, the wait sign would reappear. Participants were only allowed to press each haptic object twice. Touching a third time set off a loud beep without the visual object being shown.

Participants were allowed, even encouraged, to have breaks whenever they wanted and the experimenter asked at least every hour, whether or not the participant wanted a break.

#### 2.4.2. Procedure

**Figure 3:**
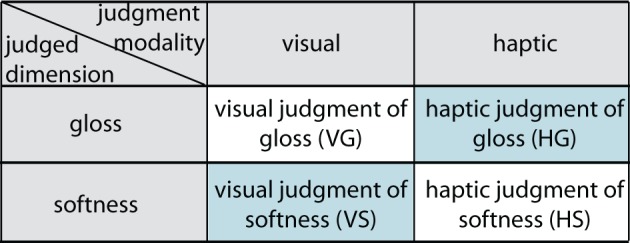
**The four experimental conditions.** In our 2 × 2 between-participants design, participants judged on one of two dimensions, gloss or softness, using one of two judgment modalities, visual or haptic. This resulted in two natural judgment task: visual judgment of gloss and haptic judgment of softness, and two novel judgment tasks: a haptic judgment of gloss and a visual judgment of softness (both in light blue).

We used a balanced 2 × 2 between-participants design and a 2-AFC task with seven participants per experimental condition. The complete experiment consisted of four experimental conditions, which are visualized in Figure 3. In each condition, an observer was asked to make a 2-AFC judgment based on a particular stimulus property (judged dimension), either glossiness or softness, using either vision or haptics as judgment modality.

We used a balanced design (each pair of stimuli was shown twice with stimuli changing sides) and trials were randomized across participants.

In all conditions, we presented a standard stimulus, defined by both a haptic and a visual cue. The type of comparison stimulus we provided was based on the judgment modality: it was either defined by a haptic compliance cue for haptic judgments or a visual gloss cue for visual judgments. Thus, when making a haptic judgment of softness (“which one felt as being softer”), observers were given a standard stimulus and a haptic-only comparison stimulus, whereas when making a visual judgment of softness (“which one looks as being softer”), they were given a standard stimulus and a visual-only comparison stimulus.

Because compliance and gloss are unrelated cues, we defined the compliance and gloss values in JND-related units (−3 … 3). The haptic cue with the lowest compliance (hardest) and the most glossy visual cue were assigned a value of −3 and the haptic cue with highest compliance (softest) and respectively the least glossy visual cue, were assigned a value of 3. This assignment was in agreement with our imposed artificial correlation (Pearson's) between increasing gloss and decreasing compliance (increasing hardness).

For each experimental condition, we ran two separate sessions, on different days. In the “initial” session, we tested for any pre-existing associations between haptic compliance and visual gloss cues and in the second session learning and testing were combined. The set of standard stimuli that we used to test for pre-existing associations was defined by the following main cue-associated cue combinations ([main cue, associated cue]): [−1, −1], [−1, 0], [−1, 1], [0, −1], [0, 1], [1, −1], [1, 0], [1, 1]. To ensure that there was no overall Pearson correlation between the two cues, we used two additional main cue-associated cue combinations: [0, −2], [0, 2]. All of these combinations were compared to all seven comparison stimuli and were repeated six times (three repetitions and presentation on each side). In addition, we added some “noise” trials in which the following standard stimuli were only compared to the three nearest (neighbors of main cue) comparisons: [3, −1], [3, 1], [−3, −1], [−3, 1]. The total session consisted of 492 (8 × 7 × 3 × 2 + 2 × 7 × 3 × 2 + 4 × 3 × 3 × 2) trials. Participants did not receive any feedback on their performance during and after this session. On average, participants needed around 2 h to complete this session.

The second session consisted of a training or learning part, and a second part in which learning and testing trials were interleaved. In the training part, we used the following main cue-associated cue combination as standard stimuli ([main cue, associated cue]): [−1, −1], [−1, −2], [1, 1], [1, 2]. These combinations were compared to all seven comparison stimuli and repeated twice (balanced design, total of (4 × 7 × 2 × 2) 56, trials). The overall Pearson's product-moment correlation coefficient in this part was 0.94. In the second part, the following standard stimuli were compared to the seven comparison stimuli a total of six times (including balancing): the test set: [−1, −1], [−1, 0], [−1, 1], [0, −1], [0, 1], [1, −1], [1, 0], [1, 1] and a “correlated cue set”: [−3, −3], [−2, −2], [0, 0], [2, 2], [3, 3]. An additional set was repeated four times: [−3, −2], [−2, −3], [−2, −1], [−1, −2], [2, 1], [1, 2], [3, 2], [2, 3]. This part consisted of 770 trials (13 × 7 × 3 × 2 + 8 × 7 × 2 × 2). The overall Pearson's product-moment correlation coefficient in this part was 0.85. Participants did not receive any feedback on their performance during either part. Note that learning and testing took part on the same day within this session. Participants needed 3 h to complete this session.

### 2.5. Analysis

**Figure 4:**
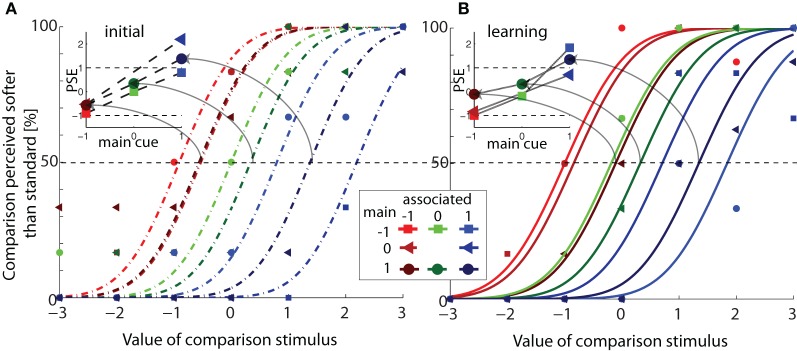
**Example of psychometric curve fits. (A)** The proportion of trials in which the comparison was perceived as softer than the standard is plotted against the softness of the comparison, for one participant in the haptic soft condition in the initial session [data and fits of the learning session are shown in panel **(B)**]. Series of data points represent the percentage of judgments that the comparison was judged as being softer than the standard separated by the different standard stimuli (different symbols and colors). Lines represent a set of cumulative Gaussian functions that was fitted to the data points. Functions share the standard deviation (as defined in the fit), but differ in mean (or PSE). In the inset, PSE values are plotted against the main cue value. PSEs of data with the same associated cue value are connected by (dotted) lines. Note that contrary to our predictions, PSEs of same main cue value did not overlap, but depended on the associated cue. For this participant, the PSE values were greater than 1 (a boundary defined by a reliability-weighted cue combination model). **(B)** Fitted psychometric curves (and actual data) of the same participant in the learning session. In the inset, PSE values are plotted against the main cue value, with PSEs of data the same associated cue value connected by lines.

From the collected (judgment) data, we calculated the proportion of trials in which the comparison was perceived as softer/less glossy than the standard per softness/glossiness value of the comparison. We then fitted cumulative Gaussian distributions to these proportional values per phase of the experiment (initial or learning) under the assumption that the JND was equal for each standard stimulus (per experimental phase). To this end, we simultaneously fitted eight cumulative Gaussian distributions to the data collected with the eight standard stimuli using eight biases (PSE) and a single standard deviation (JND) as free parameters in a least-square error fit (for an example of these fits see Figure 4). The PSE is defined as the softness/glossiness of the comparison stimulus at which discrimination performance is random (here a performance of 0.5). The 84%-discrimination threshold (JND) is defined as the difference between the PSE and the softness/gloss of the comparison when it is judged softer/matter than the standard 84% of the time. After fitting, we selected data based on the fitted JND parameter. If the JND of both the initial and the learning phase deviated more than two standard deviations from the average JND (calculated per phase), a participant was removed, because it meant that the participant displayed random behavior in both phases. With this criterion a total of three participants were removed from further analysis, resulting in one participant less in the haptic gloss, visual soft and visual gloss conditions.

To test for differences in learning between the four experimental groups, we used a MANOVA (multi-variate mixed-design general linear model, SPSS) on the estimated PSE and JND values. Even if the data do not perfectly comply to the assumptions of normally distributed data and homoscedasticity, these are robust test, meaning that deviations from these assumptions generally do lead to acceptable test results. Depending on the parameter to be tested, we used different within-participant and between-participant variables of which the details can be found in the corresponding results section. We used a significance criterion of *p* < 0.05. Where appropriate, we tested on individual groups using one- or two-sided Student's *t*-tests in order to clarify statistically significant differences between groups and/or sessions.

## 3. Results

An example of the data we fitted under the assumption that the JND was equal for each standard stimulus (per experimental phase), can be seen in Figure 4, where we plotted the proportion of trials in which the comparison was perceived as softer than the standard against the softness of the comparison, for one participant in the haptic soft condition. Data and fits of the initial phase are given in panel A, and those of the learning phase in panel B. Series of data points represent the percentage of judgments that the comparison is softer than the standard separated by the different standard stimuli (different symbols and colors). Lines represent a set of cumulative Gaussian functions that was fitted to the data points. Functions share the standard deviation (as defined in the fit), but differ in mean (or PSE).

In each inset, we plotted the PSE against the main cue for each associated cue. We predicted that in the initial phase, the PSE should correspond to the main cue value and not depend on the associated cue value, which would yield overlapping curves in the inset of panel A. Whereas after learning, the PSE should depend on both cues, if cue integration did occur, and the curves should no longer overlap (inset panel B). As predicted, the PSE values of this participant did depend on the value of the main cue, the PSE increased with increasing main cue value, see both insets. Contrary to our predictions, the PSE (of this participant) also depended on the associated cue value in both experimental phases (the curves for different associated cues did not overlap). Note that reliability-weighted cue combination should yield slopes ≤ 1, because in this theory the sum of weights should always equal 1 and the slope (in the current figure) defines the weight given to the main cue. Thus, for this participant an other type of interaction between main and associated cues is observed.

In both novel-task conditions, haptic gloss and visual soft, there were four participants (one in the visual soft condition and three in the haptic gloss condition) of whom the data fitted better to a cumulative Gaussian distribution that was tilted in the direction opposite to the one defined by the correlation between the two cues in the learning phase. The data of these mirror-model participants suggested that they, at least in the initial phase, associated an increase in compliance (softness) with an increase in gloss (whereas in the training trials we correlated an increase in compliance with a decrease in gloss).

### 3.1. Estimated points of subjective equality

**Figure 5:**
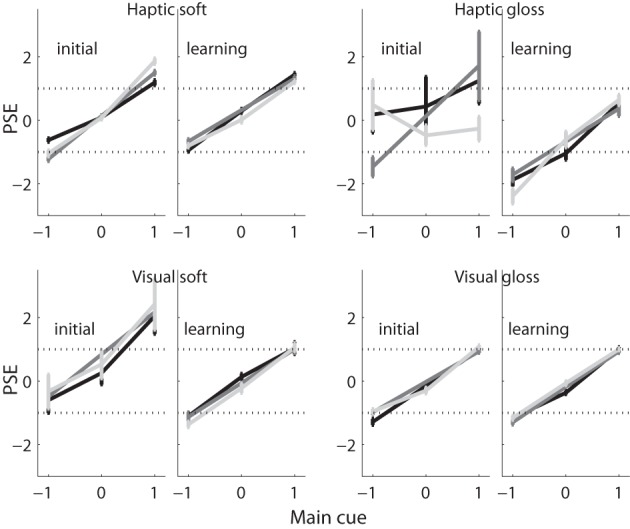
**PSEs vs. main cue.** The average PSE (and standard error) across participants are plotted against the main cue value, per experimental condition and experimental session. Values belonging to the same associated cue are connected by lines, with −1 in black, 0 in gray, and 1 in light gray.

Following the same style as that of the insets in Figure 4, we plotted the average PSE across participants with standard errors in Figure 5.

Individual PSE values entered a MANOVA with main cue (−1 vs. 0 vs. 1), associated cue (−1 vs. 1) and learning as within-participant variables, and as between-participant variables judged dimension and judgment modality. This design enabled us to separate effects of either cue. In order to get a balanced design, we did not use conditions with an associated cue value of 0. A control analysis including these stimuli yielded the same conclusions. In case of mirror-model participants, the main cues were mirrored before they entered the analysis in order to keep the direction of gloss and softness judgments the same across participants (i.e., higher/positive values meaning less glossy or softer, respectively). The associated cue was not inverted.

The overall (ANOVA) analysis showed that participants' judgments systematically depended on, and increased with, the value of the main cue, *F*_(2, 44)_ = 143, *p* ≤ 0.001. As predicted, the relation between main cue and judgment was modified by learning, *F*_(2, 44)_ = 3.5, *p* = 0.04, whereby the effect of learning was modified by the judged dimension and the judgment modality (interaction: main × learn × judged dimension: *F*_(2, 44)_ = 6.0, *p* = 0.004; interaction main × learn × judgment modality: *F*_(2, 44)_ = 4.7, *p* = 0.014; interaction main × learn × judged dimension × judgment modality: *F*_(2, 44)_ = 3.5, *p* = 0.038). There were no other reliable effects. Even though direct effects of the associated cue—as they were predicted from the weighted averaging scheme—failed to reach significance, the manifold of interaction effects with the main cue reject the possibility that participants based their judgments solely on the main cue. In that case, the participants judgments should have depended on the main cues value irrespective of learning, dimension or modality (i.e., a main cue of −1, 0, or 1 should have resulted in a judgment of −1, 0, or 1 in each and every condition). Put in other words, the results suggest influences of the double-cue situation on the judgments that depended on the interaction between learning, dimension and modality condition.

In order to clarify these differences between the four tasks, we conducted four additional analyses (MANOVA) separated by judged dimension and judgment modality (within-participant variables learning, main cue and associated cue). To make the influence of the double-cue situation better visible, we conducted these analyses on PSE data from which the to-be-expected effect of a main-cue alone strategy was eliminated (simply by subtracting the main cue value from each single PSE).

In the visual gloss condition, this analysis did not yield any significant effect. This result is consistent with the view that participants judged visual gloss on the basis of the visual main cue alone, and did so similarly before and after learning the correlation with the haptic cue. The same lack of effect was observed for the visual soft condition, even if numerical effects (see Figure 5) seem to suggest otherwise. Thus, the PSE analyses suggest that participants based their visual judgments of both gloss and softness solely on the (main) gloss cue.

In contrast, in the haptic soft condition, participants deviated from the main-cue alone strategy: a remaining effect of the main cue, *F*_(2, 14)_ = 6.8, *p* = 0.009, indicated an “over-weighting” of the main cue, i.e., judgments were spread wider apart than the main cue values. The interaction main cue × associated cue × learning, *F*_(2, 14)_ = 6.9, *p* = 0.008, indicated a more complicated modification of this effect. Additional separate analyses (MANOVA) for each learning condition (variables: main and associated cue) tracked down these deviations to the initial phase (i.e., there was no reliable effect after learning the correlation). Here, we observed a significant interaction between the main cue and the associated cue, *F*_(2, 14)_ = 6.6, *p* = 0.014 (corrected according to Huynh and Feldt, 1976), indicating that over-weighting occurred in particular with one of the two associated cues (the glossier visual cue, value of 1) and before the correlation between the two cues had been learned. Taken together, these results revealed unexpected non-linear influences of the associated cue (i.e., interaction, over-weighting), before learning the correlation, which vanished with learning. Finally, in the haptic gloss condition, we observed an interaction between the main cue and learning, *F*_(2, 10)_ = 5.6, *p* = 0.042, which—by separate analyses for the two learning conditions—could be due to an under-weighting of the main cue in the initial phase (trend: *F*_(2, 10)_ = 3.64, *p* = 0.081) combined with its over-weighting after learning (trend: *F*_(2, 10)_ = 3.6, *p* = 0.067).

Taken together, indirect influences of the associated cue on the interpretation of the main cue were observed in the two haptic conditions, but not in the two visual conditions. The observed interactions between associated and main cues were unlike predicted, of a non-linear nature including over- and under-weighting of the main cue, and statistical interactions.

Originally, we expected only linear influences of the main and the associated cue, which should have changed by learning the correlation. However, the PSE analyses revealed that learning, with regard to the overall strategy and in contrast to judgment precision (see JND results in section 3.2), resulted in the elimination of non-linear influences. In the section 3.3, we conducted an additional sensitive analysis which supports these findings.

### 3.2. Estimated just noticeable differences

**Figure 6:**
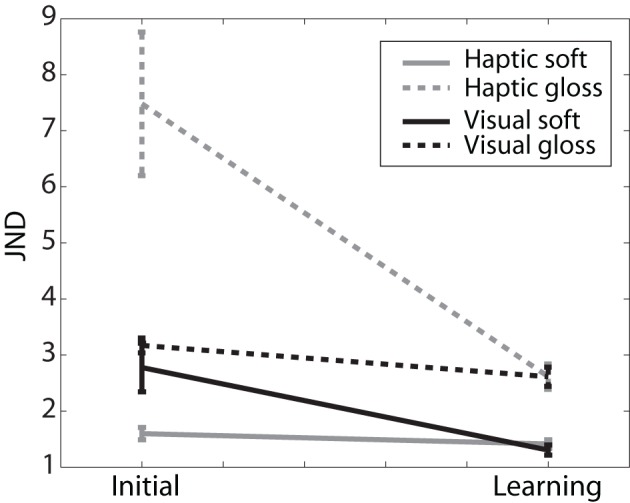
**JND.** Average JND (and standard error) across participants of initial and learning sessions for each experimental condition.

In Figure 6, the mean JND value across participants with standard error are shown for each experimental phase and condition. JND values were entered in a ANOVA (mixed-design, SPSS), with learning as a within-participants variable, and judgment modality and judged dimension as between-participants variables. There was a significant main effect of learning on JNDs, *F*_(1, 22)_ = 6, *p* = 0.025, which can be seen in the general trend of decreasing JND with learning in all experimental conditions. This effect was modified by judged dimension and judgment modality (trend: learning × judged dimension × judgment modality *F*_(1, 22)_ = 4, *p* = 0.07). In addition, we found a between-participants effect of judged dimension, *F*_(1, 22)_ = 5, *p* = 0.03.

We predicted (see introduction) that, if there was any influence of learning, the JND values should show the largest decrease in the novel tasks. Indeed, there were trends for learning in individual conditions of the novel tasks (haptic gloss *p* = 0.094, visual soft *p* = 0.12 and all novel tasks combined *p* = 0.054, 1-tailed Student's *t*-tests), but not for natural tasks (individual and combined conditions *p* ≥ 0.2, 1-tailed Student's *t*-tests). In addition, we predicted that if there would be any interaction effects with learning, these should be due to judgment modality, because the JND values could have differed between the two senses (or the two types of cues). However, there was a significant effect of judged dimension on learning (after learning: haptic gloss vs. haptic soft: *p* = 0.029, visual gloss vs. visual soft: *p* = 0.014, 1-tailed Student's *t*-test), and none of judgment modality (after learning: haptic gloss vs. visual gloss: *p* = 0.49, visual soft vs. haptic soft: *p* = 0.37, 1-tailed Student's *t*-tests). JND for gloss judgments were higher than for softness judgments both before (trend *p* = 0.073, 2-tailed Student's *t*-test) and after learning (*p* = 0.003, 2-tailed Student's *t*-test), whereas different judgment modalities were indistinguishable both before (*p* = 0.54, 2-tailed Student's *t*-test) and after learning (*p* = 0.95, 2-tailed Student's *t*-test).

Overall, these effects show that, in agreement with our prediction, JND changed with learning in the novel tasks, but not in the natural tasks. And, against our prediction, the JND after learning was independent of the judgment modality, or which cues were given to the participant, but depended on the judged dimension. Thus while given the same cues, the JND values of participants judging softness were lower than of those judging glossiness.

### 3.3. Residual analysis

As we have seen in the fitted PSE values previously, both the main and associated cue were used to make soft and gloss judgments, but they were not combined in a way congruent with a standard cue integration hypothesis (see Figure 5). Therefore, investigating changes in weight given to each cue, would not completely describe effects of learning. We therefore sought of other ways to capture learning effects. We hypothesized that by learning the arbitrary correlation, the unpredicted interaction effects should decrease. In addition, learning the association should lead to an increased linear dependence on the main and associated cues, that is cue combination instead of interaction. We decided to fit a model to the PSE values that depended linearly on the main and associated cue values. The residuals of such a fit then contain any non-linear and interaction effects of the main and associated cues on the PSE values. We used the following linear cue combination model to fit the PSE values and slightly adapted it by letting go of the constraint that the sum of weights assigned to the cues should equal 1 (as in a standard cue integration model):

$$\text{PSE}=w_{m}\times \text{main cue}+w_{a}\times \text{associated cue}$$

where *w*_*m*_ defines the weight assigned to the main cue, *w*_*a*_ defines the weight assigned to the associated cue. Both these weights were constrained between [0, 1] and we used an additional constraint that the sum of *w*_*m*_ and *w*_*a*_ should always be less than or equal to 1. These two fit parameters (*w*_*m*_, *w*_*a*_) thus give an indication of how much the PSE values linearly depended on the main and associated cue values.

The residuals of these fits now contained all the unpredicted interaction effects, such as scaling and biasing of the main cue and other interaction effects between the two cues, and they included the unpredicted variance. To separate the interaction effects from the unexplained variance, we fitted the following model to the residuals:

$$f_{\text{res}}=a_{1}\times \text{main cue}+a_{2}\times \text{associated cue}+a_{3}\times \text{main cue}\times \text{associated cue}+a_{4},$$

without any constraints on the parameters. The residuals of this fit then describe the total (or final) unexplained variance.

We now had two different measures that could show an effect of learning the association: (1) the total variance explained by the learnt-linear model (Equation 1) and (2) the part of the residuals that could be fitted by our Interaction-model [because they were due to interaction effects, Equation (2)]. Learning the arbitrary association should first of all decrease the size of the residuals due to interaction effects, because the association defined a linear non-interactive correlation between the two cues. For the same reason, it should lead to an increase in the total variance explained by the learnt-linear model.

**Figure 7:**
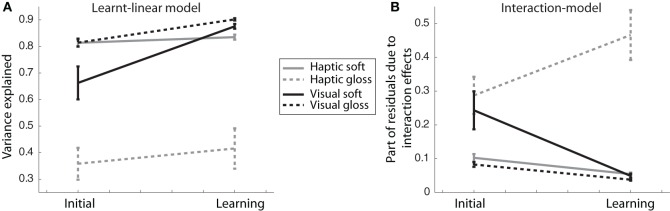
**Variance explained. (A)** Variance explained by learnt-linear model fitted to the PSEs (Equation 1). The means and standard deviations across participants are shown. **(B)** Average amount (with standard error) across participants of the residuals due to interaction effects.

The results of these fits are shown in Figure 7, with in Figure 7A, the mean variance explained by the learnt-linear model (with standard error) per experimental phase and condition and in Figure 7B, the average amount (with standard error) of the residuals that was due to interaction effects (i.e., was fitted by our Interaction-model).

We then subjected the variance explained by the learnt-linear model and the part of the residuals that were fitted by our Interaction-model to a MANOVA (multivariate mixed-design general linear model, SPSS), with learning as a within-participants variable and judgment modality and judged dimension as between-participants variables. Because these two quantities are not independent from each other, they were tested simultaneously. There was a significant main effect of learning, *F*_(2, 21)_ = 6.1, *p* = 0.008. This effect was modified by judged dimension (interaction learning × judged dimension, *F*_(2, 21)_ = 7.1, *p* = 0.004), there was only a trend for the interaction with judgment modality (interaction learning × judgment modality, *F*_(2, 21)_ = 3.0, *p* = 0.072) and a significant modification by judgment modality and judged dimension simultaneously, *F*_(2, 21)_ = 4.3, *p* = 0.028. In addition, there were significant differences between groups: a between-participants interaction effect (judgment modality × judged dimension, *F*_(2, 21)_ = 4.9, *p* = 0.018) and an effect of judged dimension, *F*_(2, 21)_ = 3.5, *p* = 0.05. We further investigated the effects of learning using 1-tailed Student's *t*-tests and the interaction effects with judgment modalities and judged dimensions using 2-tailed Student's *t*-tests.

There were at least trends for an effect of learning in both visual tasks. For the visual gloss condition, both the variance explained by the learnt-linear model (*p* = 0.023, 1-tailed) and that explained by the Interaction-model (*p* = 0.020, 1-tailed) changed with learning in the predicted directions. In the visual soft condition, there was only a trend for learning for both the variance explained by the learnt-linear model (*p* = 0.098) and that explained by the Interaction-model (*p* = 0.095).

For both haptic tasks, however, we found only a trend for learning in the Interaction-model for haptic soft (*p* = 0.091) and no significant effects of learning within the learnt-linear model (*p* = 0.32) and no effects of learning in either model for the haptic gloss condition (learnt-linear: *p* = 0.41, Interaction: *p* = 0.22).

In addition, learning had a differential effect on the two novel tasks. Whereas they did not differ before learning (learnt-linear: *p* = 0.18, Interaction: *p* = 0.8, 2-tailed Student's *t*-tests), they did so afterward (learnt-linear: *p* = 0.034, Interaction: *p* = 0.044, 2-tailed Student's *t*-tests). Both before and after learning, the variance explained by the learnt-linear model was lower for the haptic gloss condition compared to those of the two natural tasks (initial: vs. haptic soft: *p* = 0.005, vs. visual gloss: *p* = 0.013, learning: vs. haptic soft: *p* = 0.024, vs. visual gloss: *p* = 0.026, 2-tailed Student's *t*-tests) and higher for the Interaction-model only after learning (vs. haptic soft: *p* = 0.021, vs. visual gloss: *p* = 0.040, 2-tailed Student's *t*-tests). The visual soft condition, however, was not different from either natural task both before and after learning.

Taken together, these results support the previous findings of the PSE analyses, namely that learning—in agreement with the correlation—occurred in visual judgment tasks (there were trends in both visual tasks), but not in either haptic judgment task. Moreover, they showed participants performed better in the visual novel task (visual soft judgment) than in the haptic novel task—reaching the same high-level performance as in the natural visual task.

## 4. Discussion

In this study, we investigated whether learning from touch-to-vision is similar to learning from vision-to-touch. To this end, we introduced an artificial correlation between visual gloss and haptic compliance cues and investigated how learning this association influenced two natural judgments (visually judging gloss, haptically judging softness) and two novel judgments (visually judging softness and haptically judging gloss).

Our analyses of PSEs revealed unexpected non-linear interactions, whereas our sensitive analyses of explained variance revealed that learning (including de-learning of non-linear interactions) occurred, in particular, in visual tasks. In addition, the analyses of explained variance showed that “performance” after learning was better in the visual novel task (similar to “performance” in the natural visual task), than in the haptic novel task. Taken together, these results suggest that vision does not educate touch as efficiently as touch seems to educate vision.

To our knowledge, our study is the first to show that learning between the senses depends on its direction. In many previous studies, it has been shown that touch teaches vision, however, the reverse whether vision can teach touch had not been investigated thoroughly in adults, so far.

Our study is in agreement with the results from Ernst (2007) that humans have ability to learn from cue-associations and that previously unrelated cues can be recruited for a judgment task, if they are positively correlated [see also Haijiang et al. (2006) for cue recruitment in binocular rivalry tasks]. Our result that touch teaches vision is also in agreement with previous studies investigating e.g., surface orientation and depth (Ernst et al., 2000; Atkins et al., 2001; Ho et al., 2009; van Beers et al., 2011). However, we did not find any sign of reliability-weighted cue combination, as reported previously, but found cue-interaction instead. Although the difference may have occurred due to lack of learning, van Beers et al. (2011) have shown that learning to integrate haptic cues for surface slant estimation can occur quickly (with 55 trials) and within the same day. However, these time scales may apply to studies where the properties to be estimated can be sensed by both senses and not to our study in which the material properties to be estimated could only be estimated by one or the other sense.

We found that the JND depended on the judged dimension (gloss vs. soft), irrespective of the judgment modality—and thus irrespective of which cues were available for making the judgment; an effect that became clearer after learning. This may mean that participants were using different cues, or using the cues differently, in the novel tasks compared to the natural tasks. Thus, these results showed that after learning, at least in the novel tasks, estimation of the stimulus property was similar for the two senses; i.e., was the same for touch-to-vision and vision-to-touch.

In this work, we studied a kind of unconstrained basic learning. Although the cues were correlated in the learning session, participants did not receive feedback on their performance and this kind of learning could mimic learning in infants. We propose that, with our study, we tapped into the neural learning process before cue integration might occur. As a precursor to maximum-likelihood-estimated integration [see Ernst et al. (2000) for a short review], the cues both influenced the judgment, but are not yet linearly integrated. We hypothesize that given enough time, neural mechanisms related to cue integration would come to play even for seemingly arbitrary cues. However, in the case of inter-modal cue-integration, such integration is often not compulsory (Hillis et al., 2002; Gori et al., 2011) and might therefore be overshadowed by single cue effects.

Taken together, our data revealed differences in learning from-touch-to-vision and from-vision-to-touch. Learning from-touch-to-vision did occur, but not the other way around.

### Conflict of interest statement

The authors declare that the research was conducted in the absence of any commercial or financial relationships that could be construed as a potential conflict of interest.
